# The Tryptophan Metabolite Indole-3-Carboxaldehyde Alleviates Mice with DSS-Induced Ulcerative Colitis by Balancing Amino Acid Metabolism, Inhibiting Intestinal Inflammation, and Improving Intestinal Barrier Function

**DOI:** 10.3390/molecules28093704

**Published:** 2023-04-25

**Authors:** Mingfei Liu, Yuxuan Wang, Haixin Xiang, Meng Guo, Shirong Li, Ming Liu, Jingchun Yao

**Affiliations:** 1 Key Laboratory of Marine Drugs, Chinese Ministry of Education, School of Medicine and Pharmacy, Ocean University of China, Qingdao 266003, China; 2 Laboratory for Marine Drugs and Bioproducts of Qingdao National Laboratory for Marine Science and Technology, Qingdao 266237, China; 3 State Key Laboratory of Generic Manufacture Technology of Chinese Traditional Medicine, Lunan Pharmaceutical Group Co., Ltd., Linyi 276005, China; 4 Linyi Key Laboratory for Immunopharmacology and Immunotoxicology of Natural Medicine, Lunan Pharmaceutical Group Co., Ltd., Linyi 276005, China

**Keywords:** colitis, 3-IAld, anti-inflammatory, intestinal barrier

## Abstract

Ulcerative colitis (UC) has attracted much attention for its negative influence on quality of life and increased risk of colorectal cancer. Chemical and biological drugs are currently the usual treatment for UC. These drugs always induce severe side effects, or patients might become resistant to these therapies. Therefore, new therapeutic options for UC are urgently needed. In this study, we discovered the inhibitory activity of the intestinal tryptophan metabolite indole-3-carboxaldehyde (3-IAld) in dextran sulfate sodium salt (DSS)-induced UC mice by targeting the TLR4/NF-κB/p38 signaling pathway. This compound effectively protected against colon length shortening and damage induced by DSS in the colon, notably reducing the severity of inflammation. The production of inflammatory factors of TNF-α, IL-6, and IL-1β was significantly attenuated when treating with 3-IAld in vivo and vitro. This might be attributed to inhibition of the TLR4/NF-kB/p38 signaling pathway. Moreover, 3-IAld could up-regulate the expression of ZO-1 and Occludin in vivo and vitro. Meanwhile, liquid chromatography mass spectrometry (LC-MS) results showed that 3-IAld could balance the aspartate and glutamate metabolism and the lysine degradation metabolism in the serum of DSS-induced colitis mice. In conclusion, 3-IAld ameliorated the intestinal barrier dysfunction and inflammatory response in DSS-induced UC mice, balanced amino acid metabolism, and inhibited the activation of the TLR4/NF-kB/p38 signaling pathway, thereby protecting mice with colitis.

## 1. Introduction

Inflammatory bowel disease (IBD), including the two main subtypes of Crohn’s disease (CD) and ulcerative colitis (UC), is an inflammatory disorder of the intestinal tract [1]. UC is characterized by intestinal barrier dysfunction, tissue damage, and inflammatory cell infiltration. Immunosuppressive agents, amino salicylates, and corticosteroids are the main drugs used to treat UC in modern clinical medicine [2]. Although they can alleviate the clinical symptoms of patients to a certain extent, the cure rate is low, and the treatment cost is high and often leads to a variety of adverse reactions. Therefore, there is an urgent need to develop targeted, effective, and non-toxic drugs for clinical treatment of UC.

Accumulating evidence suggests that UC is closely related to the homeostasis of the intestinal epithelium [3]. A complete intestinal barrier and intestinal mucosal immunity are the prerequisites for maintaining intestinal health. Intestinal barrier function is maintained by tight junction (TJs) proteins and other molecular complexes [4]. TJs, which include claudins, Occludin, and zonula occludens-1 (ZO-1), are highly dynamic complexes that seal the space among adjacent epithelial cells and are involved in regulating the permeability of substances in the gut [5]. Defects in TJ integrity eventually lead to intestinal barrier dysfunction [6]. The invasion of pathogens or other harmful substances (such as LPS, Lipopolysaccharide) will lead to the release of a series of inflammatory cytokines and over-production of reactive oxygen species (ROS) in the intestinal tract [7]. Therefore, inhibiting inflammatory cytokines and excessive oxidative stress efficiently ameliorates UC. The toll-like receptor 4 (TLR4)/nuclear factor-κB (NF-κB) signaling pathway plays a critical role in the inflammatory response, especially the activation of macrophages in the intestinal tissue [8]. TLR4, the first TLR protein discovered, is associated with a variety of inflammatory responses and is a major receptor for lipopolysaccharide responses [9]. The interaction between TLR4 and Myeloid differentiation factor 88 (MyD88) in the TIR domain triggers a cascade of downstream signals that activate the NF-κB signaling pathway, secreting a variety of inflammatory cytokines and ultimately participating in the inflammatory response. Studies have shown that LPS binding to TLR4 leads to the activation of transcription factors, including NF-κB and mitogen-activated protein kinase p38 (MAPK p38) [10,11,12]. Activation of transcription factors leads to the production of several pro-inflammatory cytokines, such as tumor necrosis factor-α (TNF-α), interleukin-1β (IL-1β), and interleukin-6 (IL-6).

Activation of aryl hydrocarbon receptors (AhR) in the intestine promotes the secretion of IL-22 by intestinal epithelial cells and the growth of epithelial cells in the intestine, thus maintaining intestinal health [13]. Metabolite 3-Indolealdehyde (3-IAld) is a common tryptophan metabolite in the intestinal tract and has significant anti-inflammatory effects by activating AhR receptors, such as reducing chondrocyte inflammation by modulating the AhR-NF-κB signaling pathway [14], and it has also been shown to restore the integrity of the intestinal mucosa [15]. However, the exact mechanism by which 3-IAld treats UC remains unclear. In this study, we evaluated its anti-inflammatory and antioxidant activities in vitro. In vivo, we determined the anti-inflammatory effect of the compound on DSS-induced UC in C57BL/6 mice. Furthermore, metabolic disorders occur in patients with colitis, and many researchers have elucidated the potential targets and mechanisms of many drugs in the treatment of specific diseases with metabolomics strategies successfully. Therefore, in this study we used the liquid chromatography mass spectrometry (LC-MS) technique to investigate the effect of 3-IAld on the serum of DSS-induced colitis mice.

The results showed that 3-Iald could significantly repair intestinal barrier damage in UC mice and inhibit the expression of multiple inflammatory factors by inhibiting the activation of the TLR4/NF-κB/MAPK p38 signaling pathway. In addition, LC-MS results showed that 3-Iald was able to intervene in colitis mice by balancing amino acid metabolism. In conclusion, 3-Iald is expected to be a potentially effective drug for the treatment of UC.

## 2. Results

### 2.1. Structure of 3-Iald and Its Effect on Cell Viability of RAW264.7 and Caco2 Cells

**Figure 1:**
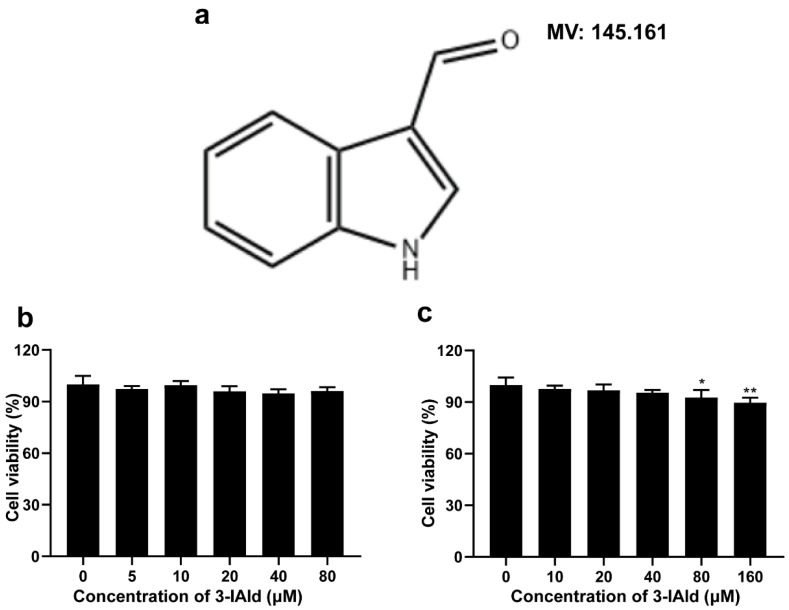
Chemical structure of indole-3-carboxaldehyde (3-Iald) and its effect on the viability of Caco2 and RAW264.7 cells. (**a**) The chemical structure of 3-Iald. (**b**) RAW264.7 was co-treated with 3-Iald (0–80 μM) for 24 h, and cell viability was assessed by CCK-8 assay. (**c**) Caco2 was co-treated with 3-Iald (0–160 μM) for 24 h, and cell viability was assessed by CCK-8 assay. Data were expressed as means ± SD (*n* = 6); * *p* < 0.05, ** *p* < 0.01 compared with 0 group.

The effect of 3-Iald on the viability of RAW264.7 and Caco2 cells was detected by CCK-8 assay. The results showed that the cell viability of RAW264.7 was 98.6, 99.2, 97.8, 97.1, and 97.9% at 3-Iald concentrations of 5, 10, 20, 40, and 80 μM, respectively, and had no effect on RAW264.7 cell viability from 0 to 80 μM (Figure 1b). In addition, the results showed that the cell viability of Caco2 was 97.7, 96.8, 95.4, 92.7, and 89.6% at 3-Iald concentrations of 10, 20, 40, 80, and 160 μM, respectively, and had no effect on Caco2 cell viability even at the high concentration of 40 μM (Figure 1c).

### 2.2. 3-Iald Inhibited the Expression Levels of NO, TNF-α, IL-6, and IL-1β Induced by LPS in RAW264.7 Cells

**Figure 2:**
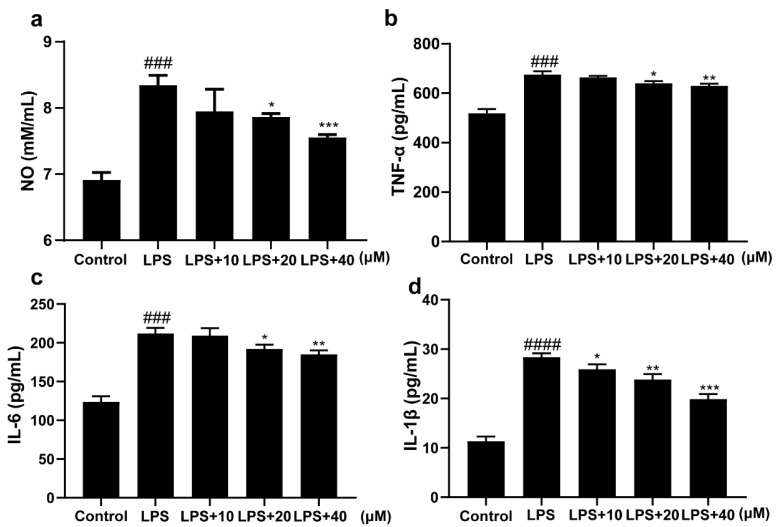
Effects of 3-Iald on LPS-induced expression levels of NO, TNF-α, IL-6, and IL-1β in RAW264.7 cells. The levels of (**a**) NO, (**b**) TNF-α, (**c**) IL-6, and (**d**) IL-1β in the cell supernatants were measured using the indicated kits according to their instructions. Data were expressed as means ± SD (*n* = 6); ^###^ *p* < 0.001, ^####^ *p* < 0.0001, compared with control group; * *p* < 0.05, ** *p* < 0.01, *** *p* < 0.001 compared with LPS group.

In order to investigate the anti-inflammatory activity of 3-Iald, the expression levels of NO, TNF-α, IL-6, and IL-1β were measured by ELISA in LPS-induced RAW264.7 cells. The results showed that LPS significantly increased the levels of NO, TNF-α, IL-6, and IL-1β in the supernatant of RAW264.7 cells compared with the normal group. Compared to the LPS group, 3-Iald co-treatment reduced the expression level of NO, and the concentrations of 20 and 40 μM have a significant difference (Figure 2b). The 3-Iald co-treatment dose-dependently reduced the expression levels of TNF-α, IL-6, and IL-1β in the supernatant (Figure 2b–d).

### 2.3. 3-Iald Reduced LPS Induced ROS Production and Up-Regulated the Expression of ZO-1 and Occludin

**Figure 3:**
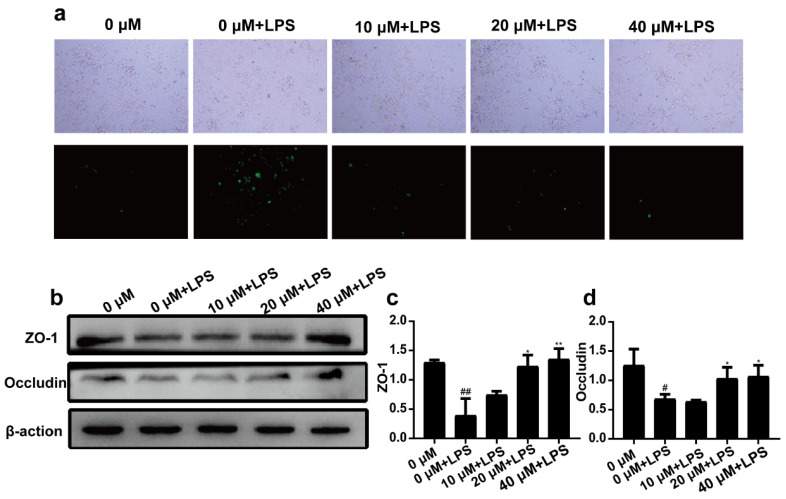
Effects of 3-Iald on LPS induced ROS production and ZO-1 and Occludin expression in Caco2 cells. (**a**) ROS levels were observed with fluorescence microscopy. (**b**) The expression levels of ZO-1 and Occludin were detected by western blotting. The relative expressions of ZO-1 (**c**) and Occludin (**d**) were analyzed by Image J. Data were expressed as means ± SD (*n* = 3); ^#^ *p* < 0.05, ^##^ *p* < 0.01, compared with 0 group; * *p* < 0.05, ** *p* < 0.01, compared with LPS group.

As shown in Figure 3a, compared with the normal group, the expression levels of ROS in the LPS group were significantly increased. However, 3-Iald co-treatment dose-dependently (10, 20, and 40 μM) reduced ROS levels. It is noteworthy that LPS could significantly down-regulate the expression of ZO-1 and Occludin in Caco2 cells. Compared with the LPS group, 3-Iald at 20 and 40 μM concentrations could significantly up-regulate the expression of ZO-1 and Occludin (Figure 3b).

### 2.4. 3-Iald Improved the Shortening of Colon Length and Pathological Damage Caused by DSS in Mice

**Figure 4:**
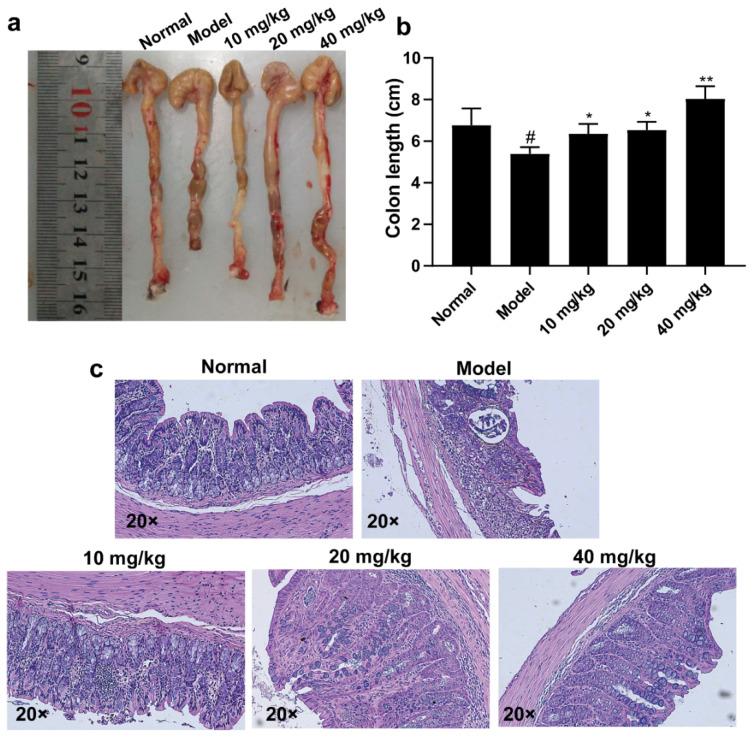
Effect of 3-IAld on colonic length and histopathology in DSS-induced UC mice. (**a**) Representative pictures of the effect of 3-IAld on the colonic length of DSS-induced UC mice. (**b**) Statistical results for each group on the effect of 3-IAld on the colonic length of DSS-induced UC mice. (**c**) Representative picture of the effect of 3-IAld on the histopathological findings in the colon of DSS-induced UC mice. Data were expressed as means ± SD (*n* = 6); ^#^ *p* < 0.05, compared with normal group; * *p* < 0.05, ** *p* < 0.01, compared with model group.

The UC mouse model was induced by free drinking of 3% DSS for seven days. As shown in Figure 4a,b, the colon length of mice was measured after sacrifice, and the colon length of the normal group was 6.77 cm, the colon length of the model group was 5.39 cm, and the colon length of 3-Iald (10, 20, and 40 mg/kg) administered mice was 6.36, 6.53, and 8.03 cm, respectively. The colons of all three 3-Iald-treated groups were significantly longer than those of the model group. Histological findings were evaluated at the end of the experiment. The results showed that the mucosal structure of mice in the model group was significantly damaged, with massive infiltration of neutrophils and almost complete loss of cupped cells. DSS-induced mucosal lesions and neutrophil infiltration were partially attenuated by moderate- or high-dose 3-IAld as assessed by microscopy, particularly in the 40 mg/kg administration group.

### 2.5. 3-IAld Alleviated the Disruption of the Colonic Barrier Caused by DSS in Mice

**Figure 5:**
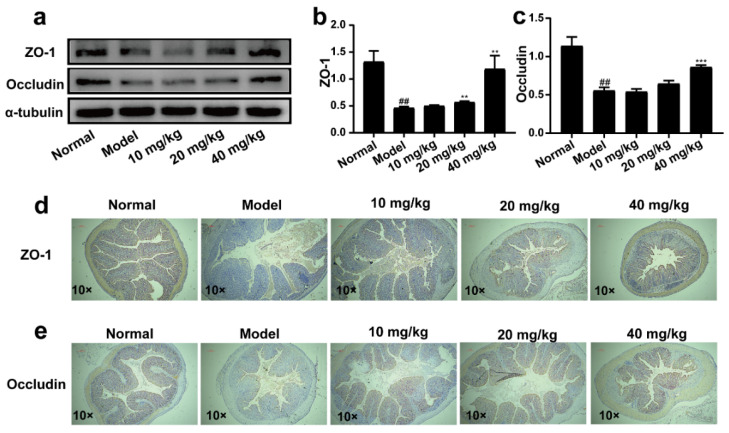
Effect of 3-IAld on ZO-1 and Occludin protein expression in the intestine of DSS-induced UC mice. (**a**) Western blotting technique to detect the effect of 3-IAld on ZO-1 and Occludin protein expression in the colon of UC mice. The relative expressions of (**b**) ZO-1 and (**c**) Occludin were analyzed by Image J. Immunohistochemical detection of the effect of 3-IAld on (**d**) ZO-1 and (**e**) Occludin protein expression in the colonic tissue of UC mice. Data were expressed as means ± SD (*n* = 3); ^##^ *p* < 0.01, compared with normal group; ** *p* < 0.01, *** *p* < 0.001, compared with model group.

In colon tissue, the structural integrity of the intestinal barrier is the basis for maintaining the normal function of the intestine. The expressions of ZO-1 and Occludin proteins were examined by western blotting. The results showed that the expression levels of ZO-1 and Occludin in colon tissues were significantly down-regulated in the model group compared with the normal group, which indicated that DSS could disrupt the barrier function of colon tissues. Compared with the model group, administration of 3-IAld (20 mg/kg) significantly improved the expression level of ZO-1 in colon tissue, but the expression level of Occludin could not be up-regulated. Administration of 3-IAld (40 mg/kg) significantly improved the expression levels of ZO-1 and Occludin (Figure 5a,b and d). The immunohistochemical results showed a significant reduction in the expression of ZO-1 and Occludin in the model group compared to the normal group. The expression of these proteins was reversed to varying degrees in each administration group compared to the model group (Figure 5d,e).

### 2.6. 3-IAld Inhibited the Expression Levels of TNF-α, IL-6, and IL-1β, and Down-Regulated the Expression Levels of TLR4, P-NF-κB, and P-P38 Proteins in DSS-Induced UC Mice

**Figure 6:**
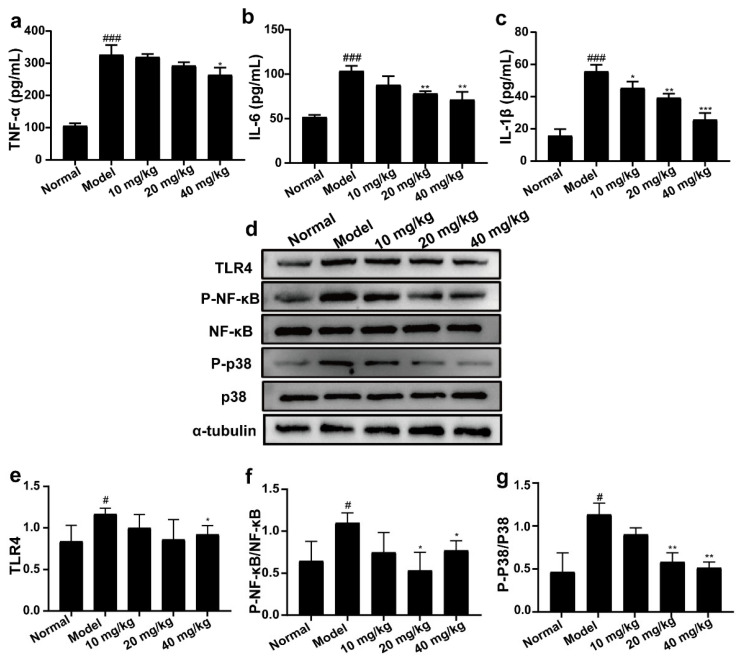
Effect of 3-IAld on the expression levels of inflammatory factors in serum and inflammation-related protein expression in intestinal tissues of UC mice. The levels of (**a**) TNF-α, (**b**) IL-6, and (**c**) IL-1β in serum of mice were measured using ELISA kits. (**d**) Western blotting technique was used to detect the protein expression levels of TLR4, P-NF-κB, NF-κB, p38, and P-p38 in the colonic tissues of UC mice. The relative expressions of (**e**) TLR4, (**f**) P-NF-κB/NF-κB, and (**g**) P-p38/p38 were analyzed by Image J. Data were expressed as means ± SD (*n* = 3); ^#^ *p* < 0.05, ^###^ *p* < 0.001, compared with normal group; * *p* < 0.05, ** *p* < 0.01, *** *p* < 0.001, compared with model group.

As shown in Figure 6a–c, the levels of TNF-α, IL-6, and IL-1β in the serum of mice in the model group were significantly increased compared with the normal group, while 3-IAld administration groups inhibited the levels of TNF-α, IL-6, and IL-1β in the serum to different degrees compared with the model group. Elevated expression of inflammation-associated proteins in colon tissues is one of the intrinsic features of UC. As expected, overexpressed protein expression was observed in DSS-treated mice. Administration with 3-IAld significantly reduced the protein levels of TLR4, P-NF-κB, and P-p38 in the colon tissues of mice.

### 2.7. Effect of 3-IAld on Serum Metabolism in DSS-Induced C57BL/6 Mice

**Figure 7:**
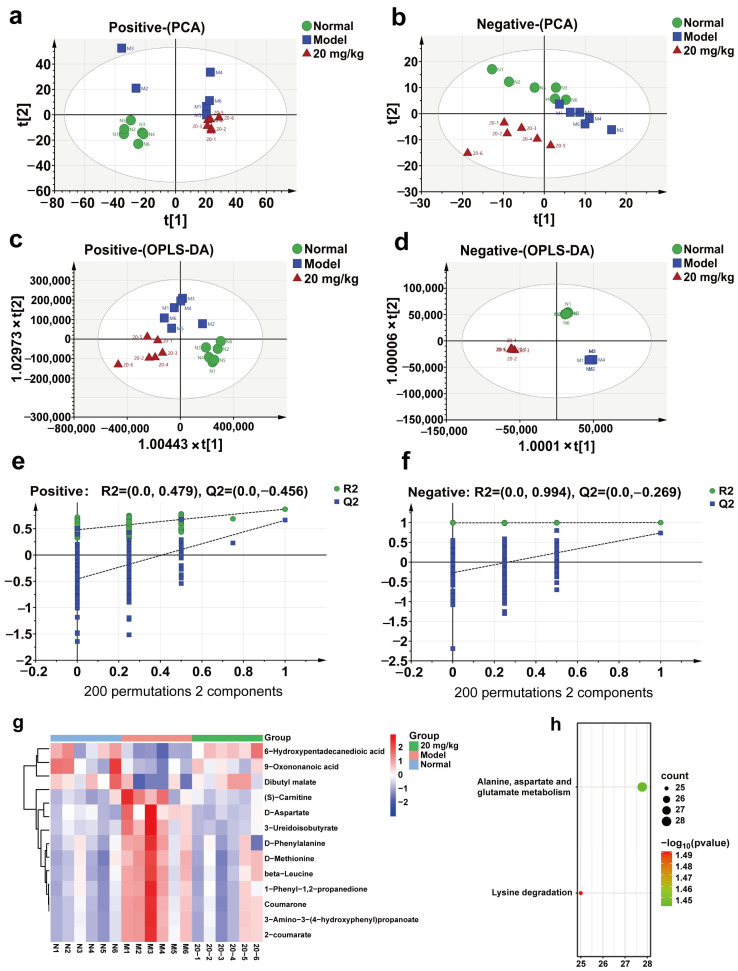
Effect of 3-IAld on serum metabolism in mice with DSS-induced colitis. (**a**) PCA analysis of serum-positive ion pattern in DSS-induced colitis mice after 3-IAld treatment. (**b**) PCA analysis of serum-negative ion pattern in DSS-induced colitis mice after 3-IAld treatment. (**c**) OPLS-DA analysis of serum-positive ion pattern in DSS-induced colitis mice after 3-IAld treatment. (**d**) OPLS-DA analysis of serum-negative ion pattern in DSS-induced colitis mice after 3-IAld treatment. (**e**,**f**) Results of orthogonal experiments in both positive and negative modes. (**g**) Heat map analysis of metabolic differentials. (**h**) Metabolic differential KEGG analysis.

To further investigate the effect of 3-IAld on the serum metabolism of DSS-induced colitis mice, we selected the 3-IAld medium-dose group (20 mg/kg) and performed an LC-MS assay on the metabolites in their serum. As shown in Figure 7a,b, the results of PCA treatment in both positive and negative ion modes showed a significant separation trend in the normal, model, and 3-IAld groups, in addition to the results of OPLS-DA treatment, which also showed a significant separation trend in the normal, model, and 3-IAld groups, indicating that 3-IAld could significantly affect the serum metabolism of DSS-induced colitis mice. The results of the orthogonal experiments showed that the metabolic data in this case were stable and reliable (Figure 7e,f). Next, we screened the compounds in each group according to (VIP > 1, *p* < 0.05); as shown in Figure 7g, 3 compounds were significantly lower and 10 were significantly higher in the model group compared with the normal group, and these 13 compounds were significantly rolled back after administration. Please see Table 1 for compound-specific information. Next, these 13 metabolic differences were substituted into the MetaboAnalyst database for metabolic pathway enrichment analysis, which showed that 3-IAld was able to affect alanine, aspartate, and glutamate metabolism and the lysine degradation metabolism pathway in the serum of DSS-induced colitis mice.

## 3. Discussion

UC is an idiopathic IBD affecting the gastrointestinal tract with lesions mainly in the colon, rectum, or the entire colorectal region. In this study, LPS-induced RAW264.7 cells, Caco2 cells, and DSS-induced colitis mice models were used to investigate the protective effects and mechanisms of specific tryptophan metabolites (3-IAld) produced by intestinal microbes on mice with colitis. The results of vitro experiments showed that 3-IAld effectively reduced LPS-induced inflammation in RAW264.7 cells and increased the protein levels of ZO-1 and Occludin in Caco2 cells pretreated by LPS. Vivo results showed that 3-IAld significantly improved the intestinal pathological damage in DSS-induced colitis mice; reduced serum inflammatory cytokines including TNF-α, IL-6, and IL-1β; improved intestinal barrier function; inhibited the activation of the TLR4/NF-κB/p38 signaling pathway in the intestine; and balanced amino acid metabolism in the serum of mice, thereby protecting mice from colitis injury.

The symptoms of UC are manifested by overproduction of inflammatory cytokines. In the study, we found that 3-IAld was able to inhibit the release of TNF-α, IL-6, and IL-1β in vivo and in vitro. These results indicate that 3-IAld has good anti-inflammatory activity. It is well known that intercellular TJs are an extremely important component of the epithelial structure that forms the intestinal barrier [3]. This barrier structure usually prevents toxic substances, such as LPS, from penetrating the intestinal cavity [16]. To date, the exact mechanism by which epithelial permeability changes has not been understood. Multiple inflammatory cytokines (such as TNF-a, IL-6, and IL-1β) secreted in the intestinal tract of UC patients may be the main cause of TJ destruction [17]. TJs play an important role in maintaining the integrity of the intestinal mucosal barrier, which is one of the strategies for the treatment of UC [18]. Treatment with 3-IAld significantly elevated the expression of Occludin and ZO-1 proteins. As we expected, these results are consistent with in vitro results. By increasing ZO-1 and Occludin proteins in the intestine of UC mice by 3-IAld, on the one hand, and by decreasing the release of inflammatory factors in the intestine, on the other hand, 3-IAld has been shown to activate the AhR receptor, which also plays a positive role in the repair of the intestinal barrier [19,20]. The TLR4 signaling pathway is one of the important mechanisms of inflammation response and a key co-recognition receptor for intestinal innate immunity [21]. The TLR4 signaling pathway is the subject of therapy (targeted inhibition) for UC [22,23,24]. In addition, NF-κB and P38 can also be activated by TLR4 to aggravate UC symptoms [10,25]. NF-κB is a nuclear transcription factor regulating overproduction of various inflammatory cytokines and adhesion molecules in a wide range of cells, thereby participating in the inflammatory reaction process. It controls the transcriptional activity of several pro-inflammatory cytokine promoters, as well as transcription factors involved in intestinal inflammation [8]. In addition, mitogen-activated protein kinase (MAPK) signaling, including p38 MAPK, plays a vital role in the modulation of inflammatory cytokines. In our experiments, we found that 3-IAld was able to reduce the expression levels of TLR4, P-NF-κB, and P-p38 proteins in the colonic tissue of UC mice, thereby inhibiting colonic inflammation, which is consistent with previous studies.

In addition, during the progression of UC, host metabolic pathways change in response to stimulation by multiple pro-inflammatory cytokines and pathogens and their metabolites, and these changes further exacerbate impaired intestinal function, leading to irreversible damage. More importantly, the onset and progression of UC are closely associated with abnormal amino acid metabolism, and amino acid supplementation can reduce inflammation and oxidative stress in inflammatory bowel disease, whereas dietary amino acid deficiency exacerbates the severity of DSS-induced colitis [25]. In the present study, 3-IAld was found to balance alanine, aspartate, and glutamate metabolism and lysine degradation in the serum of mice with DSS-induced colitis, indicating that 3-IAld can improve the symptoms of DSS-induced colitis in mice by affecting amino acid metabolism. However, this study has not yet elucidated the targets of 3-IAld in the treatment of ulcerative colitis, and its clinical trial safety and pharmacokinetic characteristics still require further in-depth research.

## 4. Materials and Methods

### 4.1. Drugs and Reagents

The 3-IAld (purity >98%) was purchased from Shanghai yuanye Bio-Technology Co., Ltd. (Yuanye Bio-Technology Co., Ltd., Shanghai, China). Antibodies against ZO-1, Occludin, TLR4, phosphorylated NF-κB, NF-κB, and phosphorylated p38 and p38 were purchased from Cell Signaling Technology (Boston, MA, USA). Antibodies against β-action and α-tubulin were purchased from Abcam Technology (Abcam Technology, Cambridge, UK).

### 4.2. Cell Lines and Cell Culture

Human colonic epithelial cells (Caco2) and mouse leukemia cells of monocyte macrophage cells (RAW264.7) were purchased from the Cell Bank of the Chinese Academy of Sciences (CAS, Shanghai, China). Caco2 cells were incubated in RPMI-1640 medium (GIBCO, Grand Island, NY, USA) supplemented with 10% fetal bovine serum (FBS) and penicillin-streptomycin (100 units/mL; Biowest, Nuaillé, France). RAW264.7 cells were incubated in Dulbecco’s Modified Eagle Medium (DMEM) (GIBCO, Grand Island, NY, USA) containing 10% FBS. The cells were maintained in an incubator containing 5% CO_2_ at 37 °C.

### 4.3. Cell Viability Assessment

RAW264.7 cells and Caco2 cells were seeded into 96-well plates at a density of 10,000 cells/well and 2000 cells/well, respectively, and placed in the cell culture incubator overnight. The 3-IAld was added and incubated with cells for 24 h, then CCK-8 (100 μL) was added into each well for another 30–60 min. Finally, the OD value was measured by microplate reader (Bio Tek, Winooski, VT, USA) at 450 nm, and the cell viability (%) was calculated.

### 4.4. Enzyme-Linked Immunosorbent Assay (ELISA)

Cells were seeded into six-well plates and incubated with different concentrations of 3-IAld for 2 h before being treated with LPS for 24 h. After treatment, the supernatant was collected and centrifugated, and the level of TNF-α, IL-6, and IL-1β was measured by using enzyme-linked immunosorbent assay (ELISA) kits (Beyotime, Shanghai, China) according to the manufacturer’s instructions. The level of TNF-α, IL-6, and IL-1β in the serum of mice was also detected using ELISA kits.

### 4.5. Intracellular ROS Assay

Caco2 cells were seeded and incubated with different concentrations of 3-IAld for 2 h before LPS (2 μg/mL) was added for 24 h. According to the manufacturer’s instructions, the cells were stained with the fluorescent marker 2,7-dichlorodihydrofluorescein diacetate (DCFH-DA) (10 μM; Sigma-Aldrich, St. Louis, MO, USA). Then the fluorescence intensity was detected by fluorescence microscopy (Olympus Corporation, Tokyo, Japan), and the level of ROS production was determined by the fluorescence level.

### 4.6. Western Blotting Assay

Western blotting assay was used to separate the lysate from isolated sterile colonic tissues or cells. In brief, the lysates were separated using 12% SDS-PAGE (sodium dodecyl sulfate-polyacrylamide gel electrophoresis) and transferred onto a polyvinylidene fluoride membrane (PVDF). Furthermore, the PVDF membrane was blocked with 5% non-fat dry milk for 1 h at room temperature and probed with primary antibodies at 4 °C overnight. Subsequently, the membrane was incubated with HRP-secondary antibody for 1 h at 37 °C. Finally, blot bands were visualized using enhanced chemiluminescence (ECL; Beyotime, Shanghai, China) reagent and captured by an imager (Chemi Scope6200; Clinx Science Instruments Co., Ltd., Shanghai, China). Finally, Image J software was used to calculate the gray value for quantitative analysis.

### 4.7. DSS Mouse Models

Male C57BL/6 mice (6–8 weeks old; weighing 19–22 g) were purchased from the National Institutes for Food and Drug Control (License Number: SCXK (Beijing) 2019-0006). Mice were housed in an air-conditioned room with the laboratory temperature maintained at 25 ± 2 °C, relative humidity of 50 ± 5%, 12 h of light/dark cycle, and access to food and water randomly. All animal experimental procedures were performed in accordance with the Guide and Use of Laboratory Animals and approved by the Laboratory Animal Ethics Committee of Lunan Pharmaceutical Group Ltd. DSS (molecular weight at 36–50 kDa) (MP Biomedicals, Morgan Irvine, CA, USA) was dissolved at 3.0% *w*/*v* with distilled water. The UC mouse model was induced by DSS for seven days followed by another three days with fresh water. Mice were given pure water and 3-IAld by gavage, and this continued until the end of the experiment. Mice were sacrificed on day 10. The colon tissues were collected and fixed in 4% paraformaldehyde solution for pathological detection for further study.

### 4.8. Hematoxylin and Eosin (H&E) Staining

The colon tissues embedded in paraffin were sliced into 3–5 μm thick sections and dehydrated via graduated ethanol series. Sections were stained with H&E. The level of inflammation and tissue damage was observed by three pathologists.

### 4.9. Immunohistochemistry (IHC)

After gradient dewaxing with xylene and ethanol, sections of colon tissue were heated in sodium citrate solution. Sections were covered with 3% hydrogen peroxide to extinguish endogenous peroxidase. PBS was then used to flush the sections three times, with the addition of 5% normal goat serum to block nonspecific binding. Next, sections were incubated with primary antibody (1:250) at 4 °C overnight. After washing with PBS three times, sections were incubated with biotin-labeled secondary antibody and streptavidin-horseradish peroxidase at room temperature, and then diaminobenzidine (DAB; Beijing Heliogod Technology Co., Ltd., Beijing, China) was added. After staining, the sections were dehydrated and sealed with a neutral resin. Target proteins were randomly observed under the microscope.

### 4.10. Analysis of The Effect of 3-IAld on Serum Metabolism in UC Mice Using the Non-Targeted Metabolomics Technique

The non-targeted metabolomics technique was used to analyze the endogenous metabolites of mice feces. In brief, a 60 mg fecal sample of each mouse was prepared, and an ice-cold mixture of methanol and water (4:1 *v*/*v*) was added. After freezing at −20 °C for 5 min, the samples were ground at 60 HZ for 2 min, and then quickly transferred to the ice bath for ultrasonic treatment for 10 min. Subsequently, fecal samples were stored at −20°C for 30 min. The samples were centrifuged at 13,000 rpm at 4 °C for 15 min, and then a 400 μL mixture of methanol and water (1:4, *v*/*v*) was added to each sample, followed by being vortexed for 30 s and then placed at −20°C for 2 h. The samples were centrifuged at 13,000 rpm at 4 °C for 10 min, and 150 μL of each sample was collected and filtered through a 0.22 μm microfilter to remove impurities. A Dionex Ultimate 3000 RS UHPLC system fitted with a Q Exactive quadrupole Orbitrap mass spectrometer equipped with a heated electrospray ionization (ESI) source (Thermo Fisher Scientific, Waltham, MA, USA) was used to analyze the metabolic profile.

### 4.11. Statistical Analysis

All data were represented as the mean ± SD (standard deviation). Comparisons between the different groups were analyzed by one-way analysis of variance (ANOVA), and a multiple comparison test was performed using the post hoc Bonferroni correction. *p* < 0.05 was considered statistically significant.

## 5. Conclusions

In summary, 3-Iald improves UC mice symptoms by reducing TLR4 expression, inhibiting NF-κB and p38 activation, attenuating histopathological changes, decreasing the expression levels of pro-inflammatory cytokines (IL-1β, IL-6, and TNF-α), increasing the expression of TJs (ZO-1 and Occludin), and balancing amino acid metabolism. Therefore, this study demonstrates that not only the reduction of inflammation and the repair of intestinal barrier are the basis for the treatment of UC, but also that the improvement of amino acid metabolic disturbance is the key to its enhanced therapeutic effect.

## Figures and Tables

**Table 1:** Details of metabolic differentials between the three groups.

No.	Name	Formula	RT (min)	Ion mod	VIP	Model vs. Normal	20 mg/kg vs. Model
1	6-Hydroxypentadecanedioic acid	C_15_H_28_O_5_	5.39	ESI−	2.29035	↓ ^##^	↑ ****
2	Dibutyl malate	C_12_H_22_O_5_	6.427	ESI−	1.2762	↓ ^#^	↑ *
3	9-Oxononanoic acid	C_9_H_16_O_3_	6.323	ESI−	1.1328	↓ ^#^	↑ *
4	beta-Leucine	C_6_H_13_NO_2_	2.513	ESI+	3.50429	↑ ^##^	↓ *
5	(S)-Carnitine	C_7_H_15_NO_3_	1.544	ESI+	1.19308	↑ ^#^	↓ *
6	1-Phenyl-1,2-propanedione	C_9_H_8_O_2_	4.946	ESI+	4.36873	↑ ^##^	↓ *
7	2-coumarate	C9H8O3	2.355	ESI+	3.97959	↑ ^##^	↓ *
8	3-Amino-3-(4-hydroxyphenyl) propanoate	C9H11NO3	2.365	ESI+	3.56817	↑ ^##^	↓ *
9	3-Ureidoisobutyrate	C_5_H_10_N_2_O_3_	1.421	ESI+	2.07972	↑ ^#^	↓ **
10	Coumarone	C_8_H_6_O	2.342	ESI+	1.73917	↑ ^##^	↓ *
11	D-Aspartate	C_4_H_7_NO_4_	1.418	ESI+	1.91917	↑ ^#^	↓ *
12	D-Methionine	C_5_H_11_NO_2_S	1.976	ESI+	2.60987	↑ ^##^	↓ **
13	D-Phenylalanine	C_9_H_11_NO_2_	4.92	ESI+	4.66763	↑ ^##^	↓ **

Data were expressed as means ± SD (*n* = 6); ^#^ *p* < 0.05, ^##^ *p* < 0.01, compared with normal group; * *p* < 0.05, ** *p* < 0.01, **** *p* < 0.0001, compared with model group.

## Data Availability

The data that support the findings of this study are available from the corresponding author upon reasonable request.

## References

[1] Bantel H., Berg C., Vieth M., Stolte M., Kruis W., Schulze-Osthoff K. (2000). Mesalazine inhibits activation of transcription factor NF-kappaB in inflamed mucosa of patients with ulcerative colitis. Am. J. Gastroenterol..

[2] Kaplan G.G. (2015). The global burden of IBD: From 2015 to 2025. Nat. Rev. Gastroenterol. Hepatol..

[3] Bai X., Bai G., Tang L., Liu L., Li Y., Jiang W. (2020). Changes in MMP-2, MMP-9, inflammation, blood coagulation and intestinal mucosal permeability in patients with active ulcerative colitis. Exp. Ther. Med..

[4] Diesing A.K., Nossol C., Danicke S., Walk N., Post A., Kahlert S., Rothkotter H.J., Kluess J. (2011). Vulnerability of polarised intestinal porcine epithelial cells to mycotoxin deoxynivalenol depends on the route of application. PLoS ONE.

[5] Turner J.R. (2009). Intestinal mucosal barrier function in health and disease. Nat. Rev. Immunol..

[6] Arrieta M.C., Madsen K., Doyle J., Meddings J. (2009). Reducing small intestinal permeability attenuates colitis in the IL10 gene-deficient mouse. Gut.

[7] Yang R., Hui Q., Jiang Q., Liu S., Zhang H., Wu J., Lin F., O K., Yang C. (2019). Effect of Manitoba-Grown Red-Osier Dogwood Extracts on Recovering Caco-2 Cells from H(2)O(2)-Induced Oxidative Damage. Antioxidants.

[8] Cui L., Feng L., Zhang Z.H., Jia X.B. (2014). The anti-inflammation effect of baicalin on experimental colitis through inhibiting TLR4/NF-kappaB pathway activation. Int. Immunopharmacol..

[9] Fukata M., Abreu M.T. (2007). TLR4 signalling in the intestine in health and disease. Biochem. Soc. Trans..

[10] Seo J.H., Lim J.W., Kim H. (2013). Differential Role of ERK and p38 on NF- kappa B Activation in Helicobacter pylori-Infected Gastric Epithelial Cells. J. Cancer Prev..

[11] Wang C., Sun H., Song Y., Ma Z., Zhang G., Gu X., Zhao L. (2015). Pterostilbene attenuates inflammation in rat heart subjected to ischemia-reperfusion: Role of TLR4/NF-kappaB signaling pathway. Int. J. Clin. Exp. Med..

[12] Ye H.Y., Jin J., Jin L.W., Chen Y., Zhou Z.H., Li Z.Y. (2017). Chlorogenic Acid Attenuates Lipopolysaccharide-Induced Acute Kidney Injury by Inhibiting TLR4/NF-kappaB Signal Pathway. Inflammation.

[13] Ngo V.L., Abo H., Maxim E., Harusato A., Geem D., Medina-Contreras O., Merlin D., Gewirtz A.T., Nusrat A., Denning T.L. (2018). A cytokine network involving IL-36gamma, IL-23, and IL-22 promotes antimicrobial defense and recovery from intestinal barrier damage. Proc. Natl. Acad. Sci. USA.

[14] Zhuang H., Li B., Xie T., Xu C., Ren X., Jiang F., Lei T., Zhou P. (2022). Indole-3-aldehyde alleviates chondrocytes inflammation through the AhR-NF-kappaB signalling pathway. Int. Immunopharmacol..

[15] D’Onofrio F., Renga G., Puccetti M., Pariano M., Bellet M.M., Santarelli I., Stincardini C., Mosci P., Ricci M., Giovagnoli S. (2021). Indole-3-Carboxaldehyde Restores Gut Mucosal Integrity and Protects from Liver Fibrosis in Murine Sclerosing Cholangitis. Cells.

[16] Guo H., Xu Y., Huang W., Zhou H., Zheng Z., Zhao Y., He B., Zhu T., Tang S., Zhu Q. (2016). Kuwanon G Preserves LPS-Induced Disruption of Gut Epithelial Barrier In Vitro. Molecules.

[17] Shi Y., Liu Z., Cui X., Zhao Q., Liu T. (2020). Intestinal vitamin D receptor knockout protects from oxazolone-induced colitis. Cell Death Dis..

[18] Wang H., Jiang Y., Li H., Wang J., Li C., Zhang D. (2020). Carbachol protects the intestinal barrier in severe acute pancreatitis by regulating Cdc42/F-actin cytoskeleton. Exp. Ther. Med..

[19] Bender M.J., McPherson A.C., Phelps C.M., Pandey S.P., Laughlin C.R., Shapira J.H., Sanchez L.M., Rana M., Richie T.G., Mims T.S. (2023). Dietary tryptophan metabolite released by intratumoral *Lactobacillus reuteri* facilitates immune checkpoint inhibitor treatment. Cell.

[20] Li Y.Y., Wang X.J., Su Y.L., Wang Q., Huang S.W., Pan Z.F., Chen Y.P., Liang J.J., Zhang M.L., Xie X.Q. (2022). Baicalein ameliorates ulcerative colitis by improving intestinal epithelial barrier via AhR/IL-22 pathway in ILC3s. Acta Pharmacol. Sin..

[21] Fukata M., Shang L., Santaolalla R., Sotolongo J., Pastorini C., Espana C., Ungaro R., Harpaz N., Cooper H.S., Elson G. (2011). Constitutive activation of epithelial TLR4 augments inflammatory responses to mucosal injury and drives colitis-associated tumorigenesis. Inflamm. Bowel Dis..

[22] Kim K.A., Lee I.A., Gu W., Hyam S.R., Kim D.H. (2014). beta-Sitosterol attenuates high-fat diet-induced intestinal inflammation in mice by inhibiting the binding of lipopolysaccharide to toll-like receptor 4 in the NF-kappaB pathway. Mol. Nutr. Food Res..

[23] Gan H.T., Chen Y.Q., Ouyang Q. (2005). Sulfasalazine inhibits activation of nuclear factor-kappaB in patients with ulcerative colitis. J. Gastroenterol. Hepatol..

[24] Luo X., Yue B., Yu Z., Ren Y., Zhang J., Ren J., Wang Z., Dou W. (2020). Obacunone Protects Against Ulcerative Colitis in Mice by Modulating Gut Microbiota, Attenuating TLR4/NF-kappaB Signaling Cascades, and Improving Disrupted Epithelial Barriers. Front. Microbiol..

[25] Hashimoto T., Perlot T., Rehman A., Trichereau J., Ishiguro H., Paolino M., Sigl V., Hanada T., Hanada R., Lipinski S. (2012). ACE2 links amino acid malnutrition to microbial ecology and intestinal inflammation. Nature.

